# LMCrot: an enhanced protein crotonylation site predictor by leveraging an interpretable window-level embedding from a transformer-based protein language model

**DOI:** 10.1093/bioinformatics/btae290

**Published:** 2024-04-25

**Authors:** Pawel Pratyush, Soufia Bahmani, Suresh Pokharel, Hamid D Ismail, Dukka B KC

**Affiliations:** Department of Computer Science, Michigan Technological University, Houghton, MI 49931, United States; Department of Computer Science, Michigan Technological University, Houghton, MI 49931, United States; Department of Computer Science, Michigan Technological University, Houghton, MI 49931, United States; Department of Computer Science, Michigan Technological University, Houghton, MI 49931, United States; Department of Computer Science, Michigan Technological University, Houghton, MI 49931, United States

## Abstract

**Motivation:**

Recent advancements in natural language processing have highlighted the effectiveness of global contextualized representations from protein language models (pLMs) in numerous downstream tasks. Nonetheless, strategies to encode the site-of-interest leveraging pLMs for per-residue prediction tasks, such as crotonylation (Kcr) prediction, remain largely uncharted.

**Results:**

Herein, we adopt a range of approaches for utilizing pLMs by experimenting with different input sequence types (full-length protein sequence versus window sequence), assessing the implications of utilizing per-residue embedding of the site-of-interest as well as embeddings of window residues centered around it. Building upon these insights, we developed a novel residual ConvBiLSTM network designed to process window-level embeddings of the site-of-interest generated by the ProtT5-XL-UniRef50 pLM using full-length sequences as input. This model, termed T5ResConvBiLSTM, surpasses existing state-of-the-art Kcr predictors in performance across three diverse datasets. To validate our approach of utilizing full sequence-based window-level embeddings, we also delved into the interpretability of ProtT5-derived embedding tensors in two ways: firstly, by scrutinizing the attention weights obtained from the transformer’s encoder block; and secondly, by computing SHAP values for these tensors, providing a model-agnostic interpretation of the prediction results. Additionally, we enhance the latent representation of ProtT5 by incorporating two additional local representations, one derived from amino acid properties and the other from supervised embedding layer, through an intermediate fusion stacked generalization approach, using an *n*-mer window sequence (or, peptide/fragment). The resultant stacked model, dubbed LMCrot, exhibits a more pronounced improvement in predictive performance across the tested datasets.

**Availability and implementation:**

LMCrot is publicly available at https://github.com/KCLabMTU/LMCrot.

## 1 Introduction

Protein crotonylation (Kcr) is an important post-translational modification (PTM) in which a crotonyl group (CH_3_CH=CHCO–) is added to lysine (K) residues on proteins, influencing their function and interaction within the cell. This PTM is associated with various cellular processes and diverse biological functions and diseases, such as cancer, neurological disorders, and cardiovascular disease (Jiang *et al.* 2021). Kcr plays crucial roles in gene expression regulation, protein stability, DNA damage repair, cell cycle progression, and more. It can occur on both histone and non-histone proteins, impacting transcription regulation and transcription–replication conflict resolution under DNA replication stress. Dynamic in nature, Kcr is regulated by writers, erasers, and readers. Its interaction with other PTMs, like ubiquitination and acetylation, is an active research field. Given its multifaceted roles in diseases, understanding Kcr can aid in targeted therapeutic development, especially for cancer (Jiang *et al.* 2021).

Identifying Kcr in proteins typically involves resource-intensive and time-consuming wet-lab experiments like high-performance liquid chromatography fractionation and high-resolution liquid chromatography–tandem mass spectrometry. In light of this, there has been a considerable increase in deep learning and machine learning research aimed at prompt prediction of Kcr sites (Ju and He 2017, Qiu *et al.* 2017, Liu *et al.* 2020). A substantial contribution in this area is Deep-Kcr, developed by Lv *et al.* (2021). This deep learning tool employs a convolutional neural network (CNN) model, combining sequence-based and physicochemical property-based features for predicting Kcr sites in HeLa cells. Another notable development is BERT-Kcr, proposed by Qiao *et al.* (2022), which leverages a pre-trained transformer called BERT (bidirectional encoder representations from transformers) to extract high-dimensional feature representations, marking the first use of language model in predicting Kcr sites. Although other NLP-based models like ELMo (Peters *et al.* 2017) and FastText (Joulin *et al.* 2016) were also explored, these models are primarily trained on natural language data, differing significantly from protein sequences. DeepCap-Kcr (Khanal *et al.* 2022) is the most recent approach in Kcr site prediction in HeLa cells, leveraging a capsule network (CapsNet) underpinned by a combination of CNNs and long short-term memory (LSTM) units. Notably, the same group has also recently introduced CapsNh-Kcr (Khanal *et al.* 2023) which is also based on CapsNet, however, the model specifically focuses on predicting Kcr sites in non-histone proteins.

Despite these advances, there are noticeable gaps. The most recent predictor, DeepCap-Kcr (Khanal *et al.* 2022) offers only a marginal improvement over its predecessor, BERT-Kcr (Qiao *et al.* 2022). Interestingly, none of these models leverage distilled representations from protein language models (pLMs). Some approaches for other PTMs employ pLMs (Pokharel *et al.* 2022, 2023a; Pakhrin *et al.* 2023), but the optimal approach for representing the site-of-interest in PTM prediction remains unclear. Although BERT-Kcr (Qiao *et al.* 2022) utilizes BERT, its sole focus on peptide sequences causes it to overlook potential global contextual information of the sites. Furthermore, no effort has been made to interpret the embeddings derived from pLMs for PTM prediction tasks. To bridge these gaps, we introduce a residual ConvBiLSTM model trained on contextualized embeddings obtained from a pLM named ProtT5. This model uses the entire protein sequence as input and learns the representation of the site-of-interest (in this case, the lysine “K” residue) by considering the embeddings of all amino acids within a window centered around the site-of-interest. By combining the global representation from ProtT5 with the conventional local peptide-based representation, which includes a supervised embedding layer and physicochemical properties, we further enhance the model’s predictive performance. Additionally, we present a comprehensive assessment of four different approaches for obtaining embeddings from the pLMs for representing the site-of-interest. Finally, by examining the attention weights and computed SHAP values, we attempt to interpret the rationale behind the superior performance of full sequence window-level ProtT5-based representation.

## 2 Materials and methods

### 2.1 Benchmark datasets

The dataset used to construct the proposed LMCrot was sourced from the work of Yu *et al.* (2020) which includes 14 311 experimentally annotated Kcr sites spanning 3734 proteins in HeLa cells. This dataset has also been utilized by recent state-of-art Kcr predictors (Lv *et al.* 2021, Khanal et al. 2022, Qiao *et al.* 2022). Following the procedures outlined in these predictors, the dataset was first subjected to a homology removal process using the CD-HIT algorithm with a dissimilarity cutoff of 0.3 (or, similarity cutoff of 0.7). This resulted in 9776 non-redundant positive sites. Subsequently, an equal number of stratified non-redundant lysine (K) residues were randomly selected from the same protein sequences to serve as negative sites. The dataset was then divided into training and independent test sets, following a 3:1 ratio based on the accession ID to ensure that no proteins overlapped between the sets, thus preventing contextual information leakage. This resulted in a training set consisting of 7353 positive and 7353 negative sites and a test set containing 2421 positive and 2421 negative sites.

To further assess the generalizability of LMCrot, we also experimented with additional datasets. First, we adopted a dataset of experimentally verified Kcr sites in non-histone proteins from CapsNh-Kcr (Khanal *et al.* 2023). This dataset, which has undergone redundancy removal, data balancing, and partitioning as part of their preprocessing steps, contains 12 262 positive and 12 262 negative samples in the training set, and 3341 positive and 3341 negative sequences in the independent test set, drawn from a total of 19 287 identified sites across 4230 proteins. Additionally, we evaluated LMCrot’s performance using a non-human dataset, specifically from tobacco plants, drawn from Sun’s work (Sun *et al.* 2017). From this dataset, we collected 2044 positive sites and negative sites each.

### 2.2 Sequence encoding

Protein sequence representation in numerical space for residue-specific predictions, such as PTM tasks, often poses challenges. Traditional approaches to PTM prediction, including crotonylation, have typically relied on feature extraction from peptide sequences around the site-of-interest (in our case, “K”) (Li *et al.* 2022, Pokharel *et al.* 2023b). This approach, however, only captures the local context of the site, overlooking potential influences from amino acids that are far apart in sequence space but are in close proximity in space due to the non-linear and folded nature of proteins. Consequently, a more comprehensive representation that encapsulates both the local and global contexts of the site is required. In response to this need, our work employs a pLMs-based representation that operates on the entire sequence, thereby capturing the global context. Simultaneously, we also utilize two peptide (or, window sequence)-based encodings—the supervised embedding layer and informative physicochemical properties—to effectively capture the local environment of the site-of-interest. Given that the optimal window size across existing Kcr predictors (Lv *et al.* 2021, Khanal *et al.* 2022, Qiao *et al.* 2022) is 31, we also adhere to this size to establish the local environment of the site.

#### 2.2.1 Protein language models

pLMs, leveraging transformer (Vaswani *et al.* 2017), are pivotal in interpreting proteins using only their primary sequence. Originally designed for NLP, these models excel in detecting intricate patterns in sequential data, creating embeddings for each protein sequence segment or token. For prediction of Kcr sites, we investigate four prominent transformer-based pLMs [ProtBert (Elnaggar *et al.* 2020), ProtT5 (Elnaggar *et al.* 2020), ESM-2 (Lin *et al.* 2022), Ankh (Elnaggar *et al.* 2023)] as embedding extractors (see Supplementary Section S2 for detailed specifications) and propose four extraction methods (FSPE, FSWE, WSPE, WSWE) to coherently represent the site-of-interest. In full sequence-based per-residue embeddings (FSPE) and full sequence-based window embeddings (FSWE), the entire sequence of maximum length *N* is the input to the pLMs. FSPE yields a L × 1 dimensional tensor representing solely the site-of-interest, while FSWE produces a L × W dimensional tensor, considering the embeddings of all residues within the designated window of the site-of-interest, where *L* is the length of the embedding per amino acid and *W* is the window size, 31 in this case. Conversely, window sequence-based per-residue embeddings (WSPE) and window sequence-based window embeddings (WSWE) also produce L × 1 and L × W dimensional tensors, respectively, with the input being a window (or peptide) sequence of length *W* instead of the full sequence. Table 1 summarizes these methods w.r.t each pLM used in this work. Note that the cross-validation experiments identified ProtT5 as the optimal pLM and FSWE as the optimal embedding extraction method, leading to the selection of FSWE-based ProtT5 embeddings for the final architecture (refer to Section 3.1).

**Table 1.** Dimensions of the input sequence and output tensors for pLMs derived from FSPE, WSPE, WSWE, and FSWE.

pLM	Input dimension		Output dimension	
	FSPE and FSWE (full sequence-based)	WSPE and WSWE (peptide-based)	FSPE and WSPE (per-reside only)	FSWE and WSWE (window residues)
ProtT5 (ProtT5-XL-UniRef50)	N×1	31×1	1024×1	1024×31
ProtBERT (ProtBERT-UniRef100)	N×1	31×1	1024×1	1024×31
Ankh (Ankh Large)	N×1	31×1	1536×1	1536×31
SeqVec (SeqVec-UniRef50)	N×1	31×1	1024×1	1024×31
ESM-2 (ESM2-T36-3B-UR50D)	1024×1	31×1	2560×1	2560×31

ESM-2 can only accept sequences of up to 1024 length. SeqVec, a BiLSTM-based pLM, has also been employed for the sake of completeness.

#### 2.2.2 Local peptide-based encoding

In addition to pLMs, we employ two encoding techniques that operate on peptide (or, *n*-mer window) sequences. The first encoding is performed by the supervised embedding layer provided by Keras which learns a dense representation of the sequence as a part of the deep-learning architecture. The input for this layer is composed of word (amino acid) indices, comprising an integer-encoded window sequence that is centered around the site-of-interest. The layer is initialized randomly and is adjusted during training *via* backpropagation. There are three salient hyperparameters of the embedding layer: the vocabulary size (input_dim or *V*), the embedding dimension (output_dim or *D*), and the input length (input_length). The input_dim was set to 23, which is based on the 20 canonical amino acids, and an additional three for any non-canonical or virtual amino acids (“X”). The input_length is equal to the size of the peptide sequence (*W*), which in our case is 31 while output_dim was determined to be 15 based on fivefold cross-validation. Therefore, the embedding layer has an output dimension of 15 × 31 (D × W).

The second peptide-based encoding leverages 1343 inherent amino acid properties and classifications extracted from the FEPS server (Ismail *et al.* 2022). For a detailed description of these features, please refer to Supplementary Section S1.

### 2.3 LMCrot architecture

The LMCrot architecture employs an intermediate fusion-based stacked generalization of three base models—T5ResConvBiLSTM, EmbedCNN, and PhysicoDNN and a meta-model that learns latent representations of the base models. The details of these base models and the meta-classifier are as follows.

#### 2.3.1 T5ResConvBiLSTM

The architecture of T5ResConvBILSTM consists of two components. The first component incorporates the 24 encoder-decoder layers of the pre-trained T5 (Raffel *et al.* 2020) (ProtT5) network. The final layer of ProtT5 yields an embedding tensor of dimension L × N (where L=1024) on each of the encoder and decoder sides, where *N* represents the length of the input sequence.

The second component employs a residual convolutional bidirectional long short-term memory (resConvBiLSTM) layer to fine-tune the ProtT5 network for the Kcr prediction task. To achieve this, the model inputs a tensor of dimension 1024 × W, where *W* denotes the window size (=31), encompassing embeddings of neighboring amino acids centered around the site-of-interest (also known as FSWE tensors). These embeddings are derived from the encoder side of the last hidden layer of ProtT5 in half-precision mode. The model then employs a series of layers to produce the classification result for the input sequence. Specifically, the architecture integrates two time-distributed 2D convolution layers each with residual connections to its previous layer, followed by a BiLSTM layer with eight units to learn the sequence context in both directions of the site-of-interest. The subsequent dense layers render the classification between Kcr and non-Kcr sites. To combat overfitting, dropout layers are used throughout the model.

#### 2.3.2 Stacked generalization

**Figure 1.**
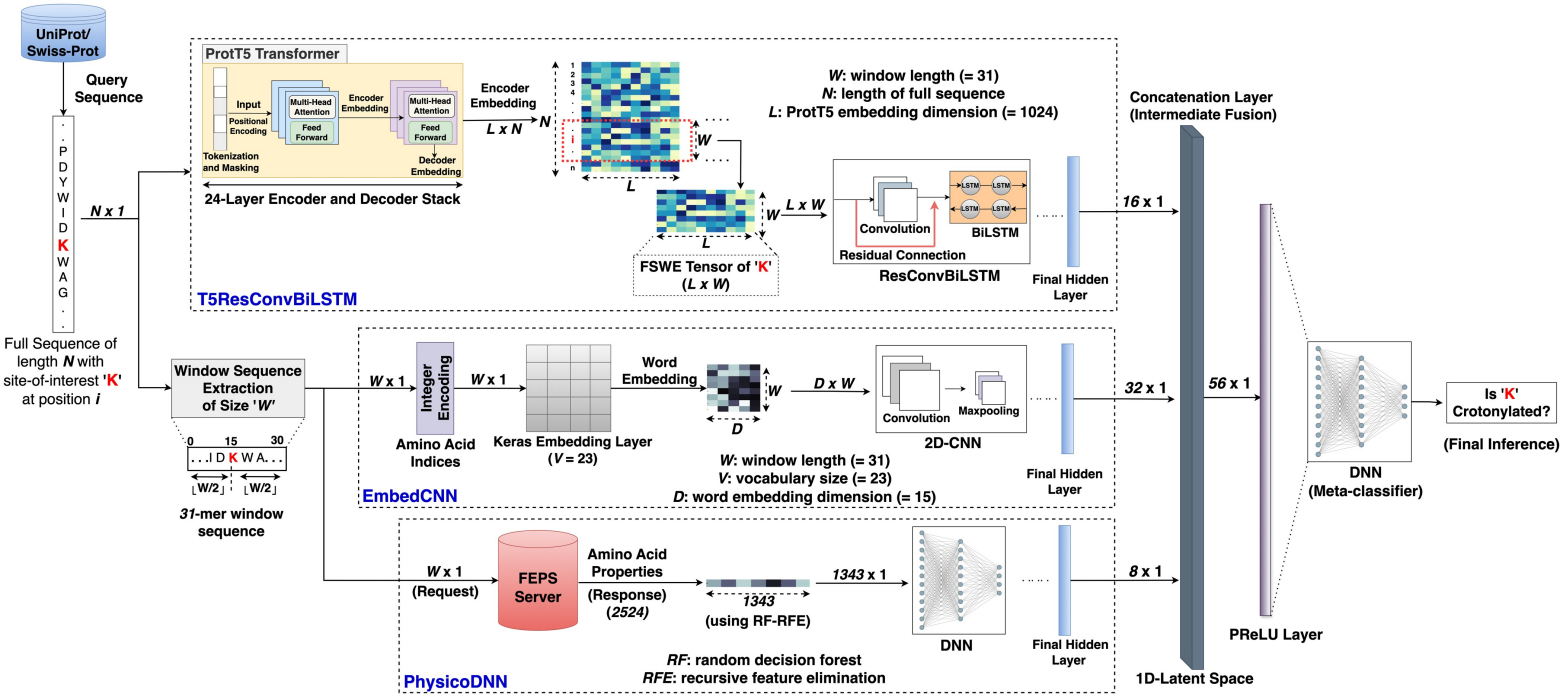
The architecture of LMCrot depicting the base models (T5ResConvBiLSTM, EmbedCNN, and PhysicoDNN) and meta-classifier. The site-of-interrogation “K” (positioned at index *i* in the input sequence of length *N*) is highlighted in bold red.

To enhance prediction robustness, the T5ResConvBiLSTM model additionally integrates the local representation of the site-of-interest using a supervised embedding layer and informative physicochemical properties. First, the embedding layer’s representation of the peptide sequence of dimension D × W (D=15 and W=31) is learned through a 2D-CNN architecture with five layers, and the respective physicochemical properties’ representation of dimension 1343 × 1 is learned *via* a three-layered DNN architecture. We dub the CNN model trained on the embedding layer as “EmbedCNN” and the DNN model on physicochemical properties as “PhysicoDNN”. Subsequently, these independently learned latent representations are fused together with the ProtT5 representation leveraging an intermediate fusion-based stacked generalization method using a three-layered DNN as a meta-classifier which produces the final classification inference of the input sequence. To this end, the features from the final hidden layers of each base model (T5ResConvBiLSTM: 16 × 1, EmbedCNN: 32 × 1, and PhysicoDNN: 8 × 1) are concatenated. These concatenated features (56 × 1 in total) are then normalized and passed through a Parametric ReLU (PReLU) layer to introduce non-linearity into the merged representation, before being fed to the meta-classifier. We term the overall stacked model as “LMCrot”. The schematic diagram of LMCrot is shown in Fig. 1.

**Figure 2.**
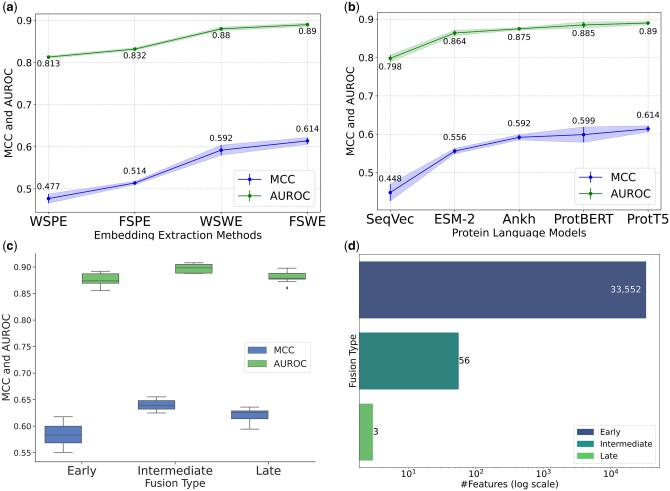
Fivefold cross-validation MCC and AUROC comparisons (a–c). (a) pLM embedding extraction methods (lineplot with one S.D.), (b) pLMs using FSWE (lineplot with one S.D.), (c) fusion types (box plot), and (d) feature size distribution of fusion types (bar graph).

The choice of intermediate fusion is driven by two primary reasons. First, late fusion might fail to capture the correlation between different representations while early fusion integrates raw ProtT5 features, which could lead to very high-dimensional input features to the meta-model (see Fig. 2d). Second, the fivefold cross-validation results corroborated the superior performance of intermediate fusion in comparison to early and late fusion methods (refer to Section 3.1).

Note that model selection for both the base models and the meta-classifier was done using fivefold cross-validation. Comprehensive details regarding the architectures of the base models and the meta-classifier are provided in Supplementary Section S3.

### 2.4 Model training and evaluation protocol

All deep-learning models were trained to minimize the binary cross-entropy loss function, with parameters initialized using the glorot uniform initializer. These parameters were optimized to reduce this loss function using the Adam optimizer with a learning rate of 0.001, with a decay rate of 0.9 for the first moment and 0.999 for the second moment. The training process was set to run for a maximum of 50 epochs, with a batch size of 512. Overfitting of the models was carefully averted using early stopping, *L*1 and *L*2 norm regularization, and monitoring the accuracy/loss curves in each fold of cross-validation. Moreover, the optimization of hyperparameters and model selection was performed using stratified *k*fold cross-validation on the training set, ensuring no overlap of proteins between the training and validation subsets of each fold. Independent testing was used to evaluate generalization error and compare our method with existing ones. For comprehensive performance assessment, metrics like Mathews correlation coefficient (MCC), geometric mean (*G*-mean), *F*1-score, area under the receiver operating characteristic curve (AUROC), and area under the precision-recall curve (AUPR) were adopted (Powers 2011) (see Supplementary Section S4). The statistical significance of our method against the other approaches was assessed using McNemar’s test and Cochran’s *Q* test (Raschka 2020).

## 3 Results

We first analyze various ML/DL architectures to identify the optimal base models for each representation, utilizing 5-fold cross-validation. Subsequently, we employ data leakage proof stacking cross-validation (Wolpert 1992) to determine the optimal model for the meta-classifier. Following this, we conduct an ablation study to assess the contribution of representations and delve into the interpretation of pLM embeddings. Finally, independent testing is performed to compare our tool with existing state-of-the-art tools, and accompanying this, significance tests are conducted, with their results exclusively detailed in Supplementary Section S5.

### 3.1 Cross-validation analysis

Using stratified fivefold cross-validation on the training set, we explored multiple pLMs (ProtT5, ProtBERT, ESM-2, Ankh, and SeqVec) and various methods (FSPE, FSWE, WSPE, and WSWE) for representing the site-of-interest across these pLMs. For per-residue embedding extraction (WSPE and FSPE), where a tensor of length L × 1 is extracted corresponding to the site-of-interest, we tested relatively simple models like DNN, SVM, RF, and XGBoost as suggested by the works of Villegas-Morcillo *et al.* (2021) and Weissenow *et al.* (2022)*.* For window embedding extraction methods (WSWE and FSWE), which produce an L × W dimensional tensors, we applied spatial and sequential models. These models, such as CNN, LSTM, BiLSM, ConvLSTM, and ConvBiLSTM, are designed to capture the spatio-temporal correlations between the embeddings of neighboring amino acids within a window.

From Fig. 2a, it is evident that FSWE secured the top rank in mean MCC and mean AUROC, closely followed by WSWE. This underlines the importance of considering neighboring embeddings (even in the case of pLM-based encoding) to boost prediction performance, rather than considering the embeddings of only the site-of-interest (WSPE and FSPE) as in prior works like LMSuccSite (Pokharel *et al.* 2022) and pLMSNOSite (Pratyush *et al.* 2023).

Figure 2b presents a sensitivity analysis using FSWE as the preferred extraction method while experimenting with various pLMs. Here, ProtT5’s superiority in terms of MCC and AUROC over other pLMs is observed. Notably, across all the embedding extraction methods, ProtT5 showcased superior performance in terms of MCC and AUROC compared to other pLMs (for a more granular breakdown for each pLM, please refer to Supplementary Table S12). In Table 2, we have delineated the performance metrics of different models using FSWE-based ProtT5 embeddings. From this table, one can discern that the ConvBiLSTM model markedly outperforms its counterparts across all evaluation metrics. Further enhancement in performance is observed when introducing residual connections into the ConvBiLSTM network (ResConvBiLSTM). Given the cumulative evidence from these analyses, we have selected the ResConvBiLSTM architecture as the most apt model and the FSWE-based extraction method to construct the ProtT5-based base model (also known as “T5ResConvBiLSTM”).

**Table 2.** Performance evaluation on fivefold cross-validation of various DL models utilizing FSWE-based ProtT5 embeddings.

Model	MCC	*G*-mean	*F*1	AUPR	AUROC
RNN	0.470	0.733	0.734	0.672	0.809
LSTM	0.500	0.748	0.754	0.684	0.829
BiLSTM	0.526	0.763	0.767	0.699	0.837
ConvLSTM	0.554	0.777	0.776	0.716	0.858
ConvBiLSTM	0.601	0.800	0.802	0.739	0.886
ResConvBiLSTM	**0.614**	**0.806**	**0.811**	**0.743**	**0.890**

The highest values are bolded in each column.

Much like the ProtT5, the optimal models for the other two base models which are trained on the embedding layer and physicochemical properties respectively were chosen based on fivefold cross-validation. Our results revealed that the 2D-CNN architecture yielded the best cross-validation performance for the embedding layer, while the DNN architecture was optimal for the physicochemical properties. Details on the cross-validation performances of various models related to these two representations are available in Supplementary Table S13. Table 3 reports the comparative performance of the base models and the final stacked generalized model based on fivefold cross-validation.

**Table 3.** Performance comparison on fivefold cross-validation between base models and stacked generalized model (LMCrot).

Base model	MCC	*G*-mean	*F*1	AUPR	AUROC
PhysicoDNN	0.562	0.780	0.786	0.717	0.861
EmbedCNN	0.585	0.788	0.786	0.733	0.883
T5ResConvBiLSTM	0.614	0.806	0.811	0.890	0.743
Stacked gen. (LMCrot)	**0.640**	**0.819**	**0.824**	**0.890**	**0.898**

The highest values are bolded in each column.

Moreover, we explored three distinct representation fusion methods for stacked generalization. As depicted in boxplot in Fig. 2c, the intermediate fusion method (merging final hidden layers) stood out, achieving the highest mean MCC and mean AUROC with a small interquartile (IQR) range (see Supplementary Table S14 for other measures). Given this performance and the rationale discussed in Section 2.3.2, we opted for the intermediate fusion-based stacking.

### 3.2 Ablation study

**Figure 3.**
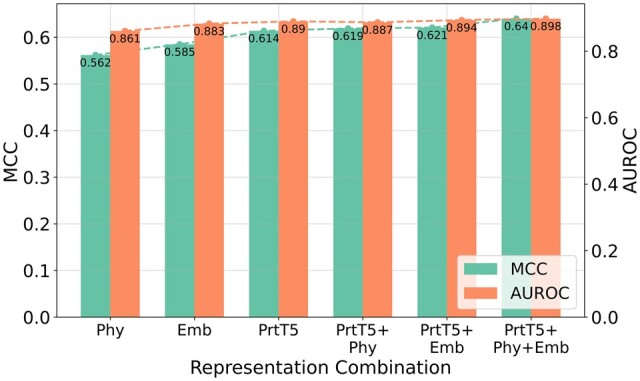
Fivefold cross-validation MCC and AUROC scores for various representation combinations. “ProtT5” is abbreviated as “PrtT5”, “Embedding layer” as “Emb”, and “Physicochemical” as “Phy”.

We sought to understand the contribution of the two additional local contextual representations when integrated with the full sequence contextual pLM. An ablation study was conducted, analyzing the mean MCC and mean AUROC based on fivefold cross-validation for each representation (see Supplementary Table S15 for other measures). Our results (shown in Fig. 3) indicate that while ProtT5 on its own surpassed the performance of the other two individual representations, a combination with either physicochemical properties or the embedding layer *via* stacked generalization offered performance enhancements. Most notably, stacking all three representations (LMCrot)—ProtT5, physicochemical properties, and the embedding layer—resulted in the most notable improvement.

**Figure 4.**
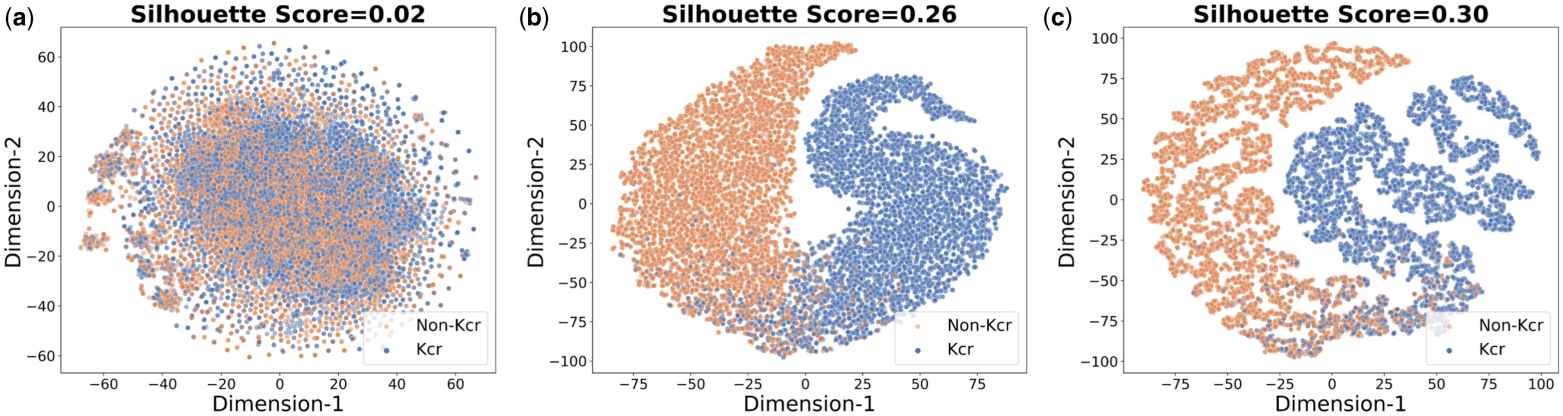
Planer t-SNE plots of (a) raw ProtT5 embeddings, (b) T5ResConvBiLSTM, and (c) stacked model (LMCrot), along with corresponding mean silhouette coefficient (or, score) (range∈[−1, 1]).

Additionally, we visualized features learned from the final hidden layer of the ProtT5 encoder, T5ResConvBiLSTM, and LMCrot using training data by projecting *t*-SNE onto a $R^{2}$ plane, configured with a perplexity of 50 and a learning rate of 200. Figure 4a displays the raw *t*-SNE of the ProtT5 embeddings, derived directly from the final hidden layer of ProtT5’s encoder, where there is a noticeable blending of the Kcr and non-Kcr datapoints with a very low Euclidean silhouette score (*S*-score) of 0.02, indicating minimal discernibility between the two. However, when we fine-tuned the pre-trained ProtT5 using the ResConvBiLSTM model on the Kcr dataset, a clearer distinction between the Kcr and non-Kcr samples emerged with an increased *S*-score of 0.26, as depicted in Fig. 4b. A notable difference was observed with a maximum *S*-score of 0.30 using the LMCrot model, which integrated local context-based features from both the embedding layer and physicochemical properties. Figure 4c shows a pronounced separation boundary and fewer datapoints overlap. This enhanced separation further asserts the benefits of combining global contextual features (from ProtT5) with local contextual features (from the embedding layer and physicochemical properties).

### 3.3 Interpretation of pLM embeddings

**Figure 5.**
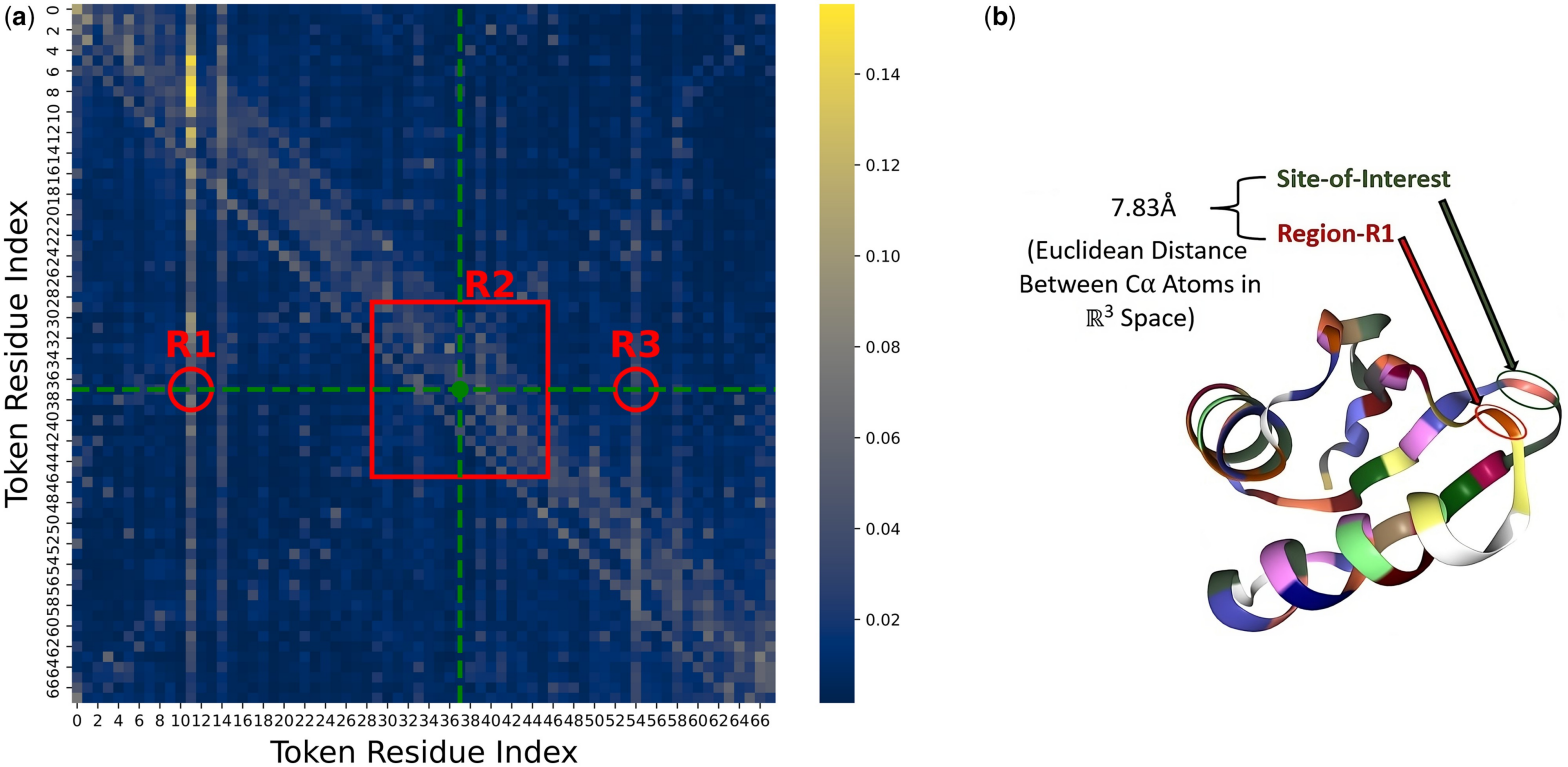
(a) Heatmap illustrating avg. attention weights for each token position in the sequence. (b) 3D structure of protein showing site-of-interest, region R1, and their Euclidean distance (in Å or $10^{-10}$ m).

First, we visualized the normalized attention weights from the final block of the ProtT5 encoder, averaged over its heads (see Supplementary Fig. S5a for individual headview), for an example protein sequence (ID: O00244). We used a heatmap to identify attention-focused regions in the embedding space (Hou *et al.* 2023). In Fig. 5a, regions R2 and R3 are within the window around the positive site (at position 37 denoted in green dot) while region R1 is outside the window. Two key observations were made: first, we can observe that adjacent embeddings (region R2) have a high association with the site whereas non-adjacent but proximal embeddings within the window (region R3) have some degree of association. This shows that considering only embeddings of the site-of-interest (in cases of WSPE and FSPE) might fail to capture the association of embeddings around the site. Second, we see an association between token position 11 (in region R1) with the token of the positive site. On referring to the 3D structure of the protein (shown in Fig. 5b), it was found that the folding of the protein brought this position spatially closer to the site-of-interest, with a euclidean distance of 7.83Å (<10Å) between the Cα atoms of the respective sites. Therefore, relying exclusively on peptide sequences (in cases of WSWE and WSPE) might fail to capture the association of distant residues affecting the site. These two observations lend support to the idea that considering window embeddings, where each embedding is generated from the entire sequence (i.e. FSWE), could be more effective, as also corroborated by cross-validation experiments.

**Figure 6.**
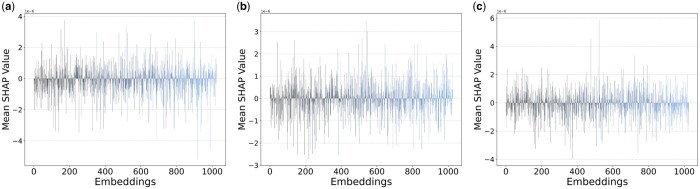
Lineplot showing mean SHAP values across all samples of ProtT5 embeddings/features (dim. = 1024) at positions (a) 15, (b) 16 (site-of-interest), and (c) 17 within the window frame of site-of-interest.

To delve deeper into understanding the impact of individual features of ProtT5 on predictions, we employed the SHAP (SHapley Additive exPlanations) method to compute the contribution values across all samples for the T5ResConvBiLSTM model, using the ‘GradientExplainer’ (expected gradients) approach (Lundberg *et al.* 2017, Hou *et al.* 2023). In Fig. 6, the visualization of mean SHAP values over total samples for each feature of ProtT5 is depicted for residues at positions 15, 16 (site-of-interest), and 17 within the context of the window frame (see Supplementary Fig. S5b for all 31 positions). A close examination of the plot pertaining to the site-of-interest in Fig. 6b reveals that among the 1024 features, certain features positively influence the prediction outcome, while others exert a negative pull. Intriguingly, the model is not solely influenced by the site-of-interest; features of adjacent amino acids also weigh in on the model’s predictions (see Fig. 6a and c).

**Figure 7.**
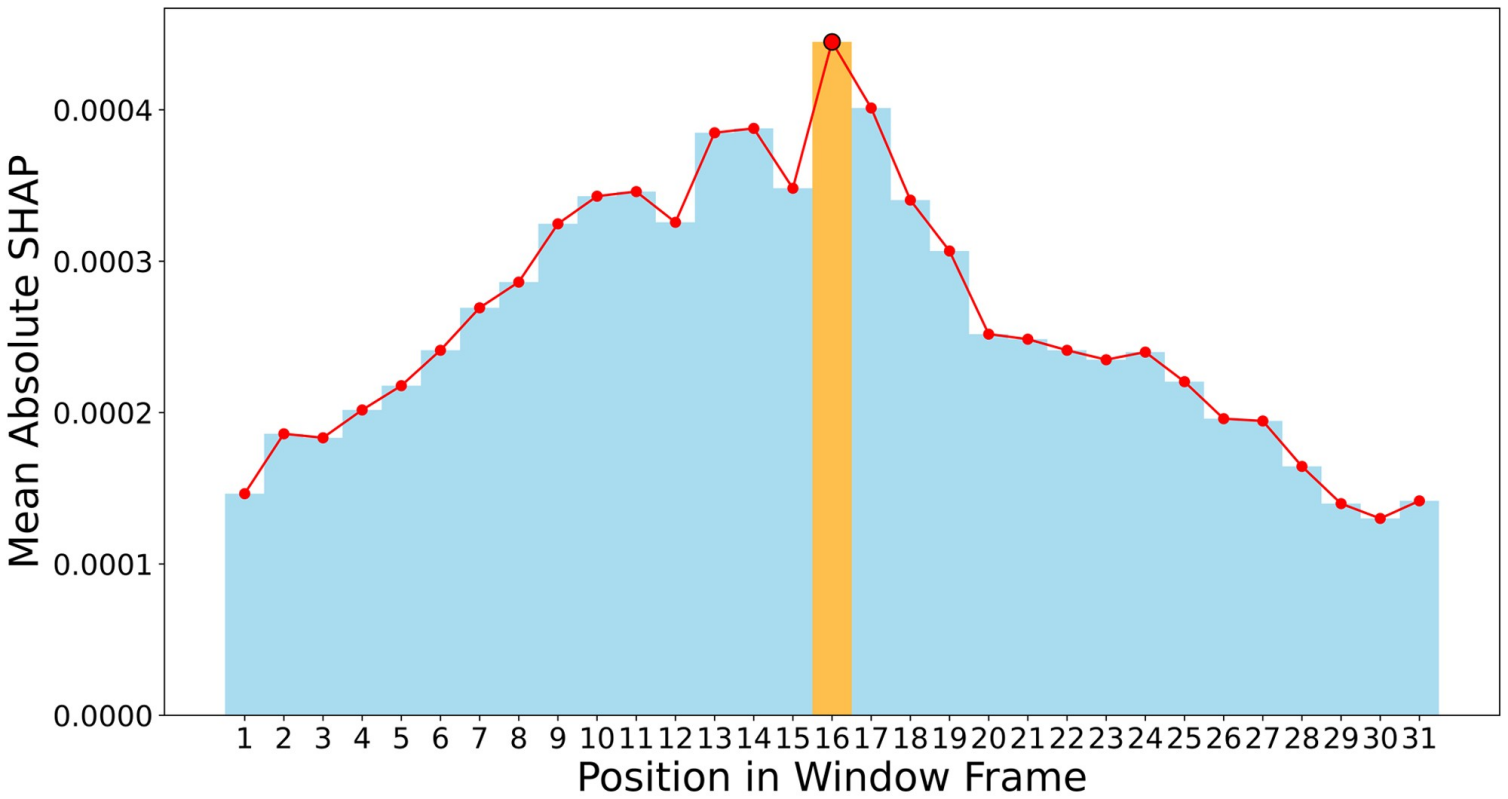
Bargraph showing mean absolute SHAP value across all features at each position in the window frame (size = 31).

Figure 7 presents a bar chart detailing the mean absolute SHAP values across all samples averaged over all the features for each position in the window frame. The site-of-interest, highlighted in orange, unmistakably stands out with the highest mean absolute SHAP value. This underscores its pivotal role in model prediction. As one moves further from this central site, the SHAP values progressively diminish, indicating a decreasing influence on the model’s predictive capability.

### 3.4 Independent testing and benchmarking

Using our independent test set (HeLa), we found that T5ResConvBiLSTM performs better than PhysicoDNN, and EmbedCNN, across all metrics (refer to Table 4). Moreover, the model utilizing stacked generalization of all three representations, aka LMCrot, demonstrated a significant improvement over those trained on individual representations (see Supplementary Section S5 for statistical tests). Notably, while LMCrot was chosen as the final predictor based on cross-validation, these independent test results underscore its standout performance compared to the base models.

**Table 4.** Performance comparison between base models and stacked model (LMCrot) on the independent test set (HeLa).

Base model	MCC	*G*-mean	*F*1	AUPR	AUROC
PhysicoDNN	0.564	0.779	0.770	0.863	0.870
EmbedCNN	0.639	0.818	0.824	0.898	0.901
T5ResConvBiLSTM	0.656	0.828	0.831	0.901	0.907
Stacked gen.(LMCrot)	**0.699**	**0.849**	**0.852**	**0.917**	**0.922**

The highest values are bolded in each column.

Next, we compared our proposed model with the existing state-of-the-art HeLa predictor, DeepCap-Kcr (Khanal *et al.* 2022). To ensure a fair comparison, we trained and tested this predictor using our training and independent test sets. As illustrated in Table 5 and ROC curve in Supplementary Fig. S6, LMCrot notably outperformed DeepCap-Kcr in all the performance measures, especially in terms of MCC with an improvement of ∼7.6%. Moreover, LMCrot achieved a more balanced performance between sensitivity and specificity, reflecting an increase in *G*-mean by ∼3.2%.

**Table 5.** Performance comparison of the existing predictor with T5ResConvBiLSTM and LMCrot on the independent test set (HeLa).

Predictor	MCC	*G*-mean	*F*1	AUPR	AUROC
DeepCap-Kcr	0.650	0.823	0.830	0.906	0.906
T5ResConvBiLSTM	0.656	0.828	0.831	0.901	0.907
LMCrot	**0.699**	**0.849**	**0.852**	**0.917**	**0.922**

The highest values are bolded in each column.

To test the generality of LMCrot across diverse datasets, we utilized the non-histone Kcr dataset from CapsNh-Kcr (Khanal *et al.* 2023) and the tobacco dataset (Sun *et al.* 2017). Initially, we trained and tested LMCrot on the CapsNh-Kcr training and testing sets. As the CapsNh-Kcr did not release the balanced test set they used, we created a balanced test set on our own and employed their model to derive results. Our observations (refer to Table 6) revealed that LMCrot significantly outperformed CapsNh-Kcr, showcasing improvements of ∼14.8%, ∼6.6%, and ∼4.6% in MCC, AUPR, and AUROC, respectively.

**Table 6.** Performance comparison of the existing predictor with T5ResConvBiLSTM and LMCrot on the non-histone test set.

Predictor	MCC	*G*-mean	*F*1	AUPR	AUROC
CapsNh-Kcr	0.589	0.786	0.807	0.833	0.862
T5ResConvBiLSTM	0.644	0.822	0.825	0.877	0.891
LMCrot	**0.676**	**0.837**	**0.842**	**0.888**	**0.902**

The highest values are bolded in each column.

Given the limited size of the tobacco dataset, we employed cross-species testing to predict all sites in this dataset using the model initially trained on the HeLa dataset. For benchmarking, we employed the DeepCap-Kcr model and observed that LMCrot surpassed its performance in all performance measures on this dataset as well (refer to Table 7).

**Table 7.** Performance comparison of DeepCap-Kcr with T5ResConvBiLSTM and LMCrot on the tobacco test set using cross-species testing.

Predictor	MCC	*G*-mean	*F*1	AUPR	AUROC
DeepCap-Kcr	0.393	0.687	0.719	0.734	0.761
T5ResConvBiLSTM	0.412	0.658	0.741	0.734	0.762
LMCrot	**0.451**	**0.695**	**0.753**	**0.749**	**0.781**

The highest values are bolded in each column.

Furthermore, comparing the difference in performance of LMCrot against DeepCap-Kcr and CapsNh-Kcr on these datasets using McNemar’s test revealed *P*-values lower than the significance level (α = .05) across all comparisons, highlighting that LMCrot’s performance is statistically significant when contrasted with these existing approaches (see Supplementary Section S5). These findings affirm that LMCrot is one of the most effective predictors for protein Kcr sites. It is also worth highlighting that the pLM-based model, T5ResConvBiLSTM, on its own, delivered better results than the existing predictors in all three datasets (see Tables 5–7). Interestingly, T5ResConvBiLSTM has ∼23.8% fewer trainable parameters than DeepCap-Kcr (see Supplementary Fig. S7). These evidences point to the notable performance of LMCrot being primarily driven by the rich representations from a pLM.

## 4 Conclusion

Protein Kcr has emerged as an essential PTM due to its crucial role in a myriad of physiological and pathological processes. In recent years, the adoption of pLM-based methodologies has seen a significant increase in various bioinformatics tasks. However, the optimal utilization of these embeddings for solving per-residue prediction problems, such as PTM prediction, is still an active field of research.

In this research, we meticulously explored various strategies to employ embeddings from pLMs, aiming to establish a reliable representation of Kcr and non-Kcr sites. Our investigation revealed that utilizing the full sequence as input to pLMs, in contrast to the traditional approach of using peptide sequences, and considering the embeddings of all amino acids within the window frame for modeling, rather than just the site-of-interest [as seen in pLMSNOSite (Pratyush *et al.* 2023), LMSuccSite (Pokharel *et al.* 2022), and Chandra *et al.* (2023)], yielded optimal results. Exploiting this methodology, termed FSWE, we developed the T5ResConvBiLSTM model, incorporating the ProtT5 pLM, which exhibited a promising performance. By merging the ProtT5 representation with the conventional peptide-based representations, namely the supervised embedding layer and physicochemical properties, through an intermediate fusion-based stacked generalization approach, we proposed LMCrot, a more robust Kcr site prediction tool. Based on rigorous independent testing on three datasets, LMCrot demonstrated superior predictive performance compared to existing state-of-the-art tools. The elevated performance is predominantly attributed to the globally contextualized representation derived from pLM and the innovative approach utilized to secure the embeddings of the site-of-interest, as substantiated by the ablation study and independent test results. By analyzing the attention weights derived from the ProtT5 encoder and the SHAP values of their corresponding embeddings, we offered insights into why it is essential to consider the entire sequence context and the embeddings of all amino acids within the window for pLM-based representation.

LMCrot, substantiated by empirical results, stands out as a promising instrument for predicting Kcr sites in proteins and is accessible in our public repository for the scientific community (https://github.com/KCLabMTU/LMCrot). The approach used in LMCrot for sequence representation can be extrapolated to numerous other PTM prediction tasks and various other per-reside prediction tasks. While LMCrot showcases promising capabilities, incorporating the structural information extracted from 3D structures of proteins can amplify the predictive accuracy.

## Author contributions statement

P.P. and D.B.K. conceived and designed the experiments; P.P. performed all the analysis and experiments, S.B. implemented the existing works, S.P., S.B., and H.D.I. tested all the programs. P.P., H.D.I., and S.P. developed the standalone version and web server. D.B.K oversaw the overall project.

## Supplementary Material

btae290_Supplementary_Data

## Data Availability

The data underlying this article are available in https://github.com/KCLabMTU/LMCrot.
