# Supplementary material for: LMCrot: an enhanced protein crotonylation site predictor by leveraging an interpretable window-level embedding from a transformer-based protein language model
Source: Bioinformatics. 2024 Apr 25;40(5):btae290. doi: 10.1093/bioinformatics/btae290 (PMC11088740; doi:10.1093/bioinformatics/btae290)
Supplement: btae290_Supplementary_Data [file btae290_supplementary_data.pdf]

LMCrot: An enhanced protein crotonylation site predicter by leveraging an interpretable window-level embedding from a transformer-based protein language model

Pawel Pratyush [1], Soufia Bahmani[1], Suresh Pokharel[1], Hamid D. Ismail[1], and Dukka B. KC[1,*]

[1] Department of Computer Science, Michigan Technological University, Houghton, 49931, Michigan, United States
* Corresponding author. dbkc@mtu.edu

Supplementary Sections

Section S1. Features derived from amino acid properties

The second peptide-based encoding, which we used, relies on features derived from the physical and physicochemical properties of the amino acids, amino acid classes, composition, distribution, and transition of amino acids on the proteins. These encodings were obtained from the Feature Extraction from Protein Sequences (FEPS) server[1] by submitting peptide sequences as requests, with each submission returning a vector of length 2524 as a response. Subsequently, feature selection was performed by employing a wrapper method called Recursive Feature Elimination (RFE)[2] on top of a Random Forest (RF) based on Gini impurity. Consequently, the initial feature set was narrowed down from 2524 to a total of 1343 important features. The complete list of feature types and their count is shown in Table S1.

Table S1. The list of the property-based feature types and numbers.

S.No	Features	#
i	Amino acid properties (Mass, #Atoms, Volume, Hydropathy Index)	120
ii	Physicochemical from AAIndex database (12 properties)	341
iii	Amino acid classes (Non-polar; Aromatic; Polar uncharged; Positively charged)	4
iv	CTD (Composition, Transition, Distribution)	147
v	Z-scale	155

vi	Sliding window amino acid Composition (SWAAC)	540
vii	Hydrophobicity and Polarity	2
viii	Multi-label amino acid classification based on overlapped properties	4
ix	ACH (Average Cumulative Hydrophobicity): Jain (JA) and Miyazawa (MI) Indexes	30
	**Total number of features**	**1343**

More details on each of the feature sets are as follows:

(i) The features (120 features) derived from the physical properties of amino acids include the amino acid mass (or, molecular weight), number of atoms in the residue, and volume of the residue (shown in Table S2).

Table S2. Amino acid properties (residue, mass, atoms, volume, hydropathy index)

S.No.	Residue	Mass (in Dalton or Da)	#Atoms	Volume (in Cubic Angstrom or $Å^3$)	Hydropathy Index
1	A	89	13	88.6	1.8
2	R	174	26	173.4	-4.5
3	N	132	17	114.1	-3.5
4	D	133	16	111.1	-3.5
5	C	121	14	108.5	2.5
6	Q	146	20	143.8	-3.5
7	E	147	19	138.4	-3.5
8	G	75	10	60.1	-0.4
9	H	155	20	153.2	-3.2
10	I	131	22	166.7	4.5
11	L	131	22	166.7	3.8
12	K	146	24	168.6	-3.9
13	M	149	20	162.9	1.9
14	F	165	23	189.9	2.8
15	P	115	17	112.7	-1.6
16	S	105	14	89	-0.8
17	T	119	17	116.1	-0.7
18	W	204	27	227.8	-0.9
19	Y	181	24	193.6	-1.3
20	V	117	19	140	4.2

(ii) The physicochemical properties' features (341 features) are derived from ten physicochemical property indexes obtained from the AAindex database (presented in Table S3).

Table S3. Physicochemical property indices of amino acids and their description.

S.No	AAindex	Description
1	TSAJ990101	Volumes including the crystallographic waters using the ProtOr
2	MAXF760101	Normalized frequency of Alpha helix (α-helix)
3	NAKH920108	AA composition of MEM of multi-spanning proteins
4	BLAM930101	α-helix propensity of position 44 in T4 lysozyme
5	BIOV880101	Information value for accessibility; average fraction of 35%
6	CEDJ970104	Composition of amino acids in intracellular proteins
7	NOZY710101	Transfer energy, organic solvent/water
8	KLEP840101	Net charge
9	NAKH900109	AA composition of membrane proteins

10	LIFS790101	Conformational preference for all beta-strands (β-strands)

(iii) Four features based on amino acid classes categorize amino acids into four classes (Nonpolar aliphatic, aromatic, polar uncharged, and positively charged).

(iv) Composition, transition, and distribution (CTD) feature (147 features) characterize the amino acid distribution patterns of a typical structural or physicochemical property in a peptide sequence.

(v) Zscale features (155 features), in which each amino acid is characterized by five physicochemical properties as listed by Sandberg *et. al.*[3].

(vi) Sliding window amino acid Composition (SWAAC), which calculates the AAC based on the sequence window of five residues sliding from the N- to C-terminus of each protein sequence (540 features).

(vii) Features based on whether a residue is hydrophobic or polar (two features), where the hydrophobic amino acids are G, A, V, L, I, P, F, M, and W while the polar amino acids are T, C, N, Q, and Y.

(viii) Features based on the overlapping properties (OP) of amino acids (four features). The OP is a multi-label classification of amino acids; each residue can be assigned one or more labels of the ten classes: polar, positive, negative, charged, hydrophobic, aliphatic, aromatic, small, tiny, and proline (shown in Table S4). However, only four properties are found important: positive, negative, tiny, and proline (four features).

Table S4. The overlapping properties of amino acids.

S.No	Class	Amino acids
1	Polar	N, Q, S, D, E, C, T, K, R, H, Y, W
2	Positive	K, H, R
3	Negative	D, E
4	Charged	K, H, R, D, E
5	Hydrophobic	A, G, C, T, I, V, L, K, H, F, W, Y, M
6	Aliphatic	I, V, L
7	Aromatic	F, Y, W, H
8	Small	P, N, D, T, C, A, G, S, V
9	Tiny	A, S, G, C
10	Proline	P

(ix) Average Cumulative Hydrophobicity (ACH) features (30 features) are based on two AAindex hydrophobicity indexes[4] as shown in Table S5. The ACH of hydrophobicity is calculated by averaging the hydrophobicity of the residues of the possible sub-windows in which the targeted lysine (K) is always in the middle. For a window of 31 residues, there will be 15 features representing the ACH of the 15 sub-windows.

Table S5. The amino indexes used for computing ACH.

JA (Jain)				MI (Miyazawa)			
**Amino acid**	**Index**	**Amino acid**	**Index**	**Amino acid**	**Index**	**Amino acid**	**Index**
A	0.06	L	1.54	A	0.4	L	1.91
R	-0.32	K	-1.07	R	-0.15	K	-0.74
N	0.13	M	0.49	N	-0.37	M	2.14
D	-0.43	F	2.48	D	-0.43	F	2.18
C	0.55	P	0.44	C	1.65	P	-0.29
E	-0.72	S	0.07	E	-0.4	S	-0.19
Q	0.46	T	0.16	Q	-0.29	T	0
G	0	W	2.81	G	0	W	1.52
H	0.03	Y	1.84	H	0.3	Y	0.68

I	1.54	V	0.97	I	2.08	V	1.51

Section S2. Description of transformer-based pLMs used in this work

Let *L* be the length of the embedding vector per residue (also called "last hidden state").

Table S6. Description of transformer-based pLMs used in this study.

pLM	Based Transformer Architecture	#Parameters	Dataset for Pre-training (#sequences in millions or M)	#layers	#Attention Heads	Training Objective	Embedding Dim. per residue (*L* x 1)
ProtT5	T5	3 B	Uniref50 (45 M)	24	32	MLM (Masked Language Modelling)	1024 x 1
ESM-2*	RoBERTa	3 B	Uniref50 (45 M)	36	-		2560 x 1
ProtBERT	BERT	420 M	Uniref100 (217 M)	24	16		1024 x 1
Ankh (large)	ConvBERT	1.2 B	Uniref50 (45 M)	48	16		1536 x 1

Note: * Sequences longer than ESM-2's 1024 length limit were truncated. For sites within the first or last 1024 residues, only these segments were used. If a site was central, a 1024-residue window around it was selected.

Section S3. Details of architectures of base models and meta-classifier

I) T5ResConvBiLSTM:

```
Layer (type)                     Output Shape         Param #     Connected to
==================================================================================================
input_layer (InputLayer)         [(None, 31, 1024)]   0           []

reshape_layer (Reshape)          (None, 31, 32, 32,   0           ['input_layer[0][0]']
                                 1)

conv2d_td_1 (TimeDistributed)    (None, 31, 32, 32,   104         ['reshape_layer[0][0]']
                                 4)

conv2d_td_2 (TimeDistributed)    (None, 31, 32, 32,   5           ['conv2d_td_1[0][0]']
                                 1)

add_1 (Add)                      (None, 31, 32, 32,   0           ['conv2d_td_2[0][0]',
                                 1)                                'reshape_layer[0][0]']

conv2d_td_3 (TimeDistributed)    (None, 31, 32, 32,   20          ['reshape_layer[0][0]']
                                 2)

dropout_td_1 (TimeDistributed)    (None, 31, 32, 32,   0          ['add_1[0][0]']
                                 1)

add_2 (Add)                      (None, 31, 32, 32,   0           ['conv2d_td_3[0][0]',
                                 2)                                'dropout_td_1[0][0]']

dropout_td_2 (TimeDistributed)    (None, 31, 32, 32,   0          ['add_2[0][0]']
                                 2)

flatten_td (TimeDistributed)     (None, 31, 2048)     0           ['dropout_td_2[0][0]']

bi_lstm (Bidirectional)          (None, 31, 16)       131648      ['flatten_td[0][0]']

flatten (Flatten)                (None, 496)          0           ['bi_lstm[0][0]']

dense_1 (Dense)                  (None, 16)           7952        ['flatten[0][0]']

dropout (Dropout)                (None, 16)           0           ['dense_1[0][0]']

output_layer (Dense)             (None, 1)            17          ['dropout[0][0]']

==================================================================================================
Total params: 139,746
Trainable params: 139,746
Non-trainable params: 0
```

- **Input Layer:** It accepts the input with a shape (window_size x 1024). The 'window_size' is the length of the window sequence (=31), and 1024 is the feature dimension of residue in the sequence.
- **Reshape Layer:** This reshapes the input to (window_size, 32, 32, 1), preparing it for the convolutional operations. It suggests that each element in the sequence is treated as a 32x32 grid with 1 channel.
- **First TimeDistributed Conv2D Layer**: Applies a convolution operation with 4 filters of size 5x5 to each sequence element individually. TimeDistributed is used to apply the same convolution operation across the time dimension (sequence).
- **Second TimeDistributed Conv2D Layer**: It has one filter of size 1x1. It serves to align the number of filters with the output from the previous convolutional layer with the residual connection. Technically, this layer performs no significant operation (except for aligning the residual connection), which is why we chose to omit it in the manuscript, referring to the model as having two convolutional layers rather than three.
- **First Add Layer**: This creates a residual connection by adding the previous layer's output to the reshaped input, which can help train deeper networks by allowing gradients to flow through the network more effectively.
- **First TimeDistributed Dropout Layer**: It applies dropout with a rate of 0.5 to each sequence element
- **Third TimeDistributed Conv2D Layer**: A second convolution operation is applied which uses 2 filters of size 3x3.
- **Second Add Layer:** Adds the output of the second convolutional operation to the output of the previous dropout layer, forming another residual connection.
- **Second TimeDistributed Dropout Layer:** Another dropout layer that applies the dropout regularization with a rate of 0.5 to the output of the previous residual block.
- **TimeDistributed Flatten Layer:** Flattens the output of the previous layers to a single vector per sequence element, making it suitable for input to the recurrent layers.
- **Bidirectional LSTM Layer:** This layer has 8 units.
- **Flatten Layer:** This layer flattens the outputs of the bidirectional LSTM layer into a 1D vector.
- **Dense Layer:** A fully connected layer that projects the flattened output down to a 16-dimensional space and introduces non-linearity with the ReLU activation function.
- **Dropout Layer**: Another dropout layer with a rate of 0.5 is added here.
- **Output Dense Layer:** A dense layer with a single unit and a sigmoid activation function.

II) EmbedCNN:

```
_________________________________________________________________
 Layer (type)                Output Shape              Param #
=================================================================
 embedding_162 (Embedding)   (None, 31, 15)            345

 lambda_162 (Lambda)         (None, 31, 15, 1)         0

 conv2d_162 (Conv2D)         (None, 16, 12, 64)        4160

 dropout_162 (Dropout)       (None, 16, 12, 64)        0

 max_pooling2d_158 (MaxPooli  (None, 4, 3, 64)         0
 ng2D)

 flatten_159 (Flatten)       (None, 768)               0

 dense_321 (Dense)           (None, 32)                24608

 dense_322 (Dense)           (None, 1)                 33

=================================================================
Total params: 29,146
Trainable params: 29,146
Non-trainable params: 0
_________________________________________________________________
```

- **Embedding Layer:** embedding layer with dimension=15, vocabulary size= 23 and input_length=31
- **Lambda Layer:** The lambda function used here is 'K.expand_dims(x, 3)', which expands the dimensionality of the input by adding a single-dimensional axis at position 3.

- **Conv2D Layer:** This layer applies 64 filters to the input data using a kernel size of (16x4). The activation function is ReLU.
- **Dropout Layer:** dropout layer with a rate of 0.3
- **MaxPooling2D Layer:** The pooling operation is performed over a 4x4 window, further reducing the size of the feature maps.
- **Flatten Layer**: This layer flattens the input from a multi-dimensional tensor to a one-dimensional tensor to be used in the following dense layers.
- **Dense Layer:** The first dense layer has 32 units with ReLU activation.
- **Dense Layer**: The final dense layer has 1 unit with a sigmoid activation function.

III) PhysicoDNN:

Layer (type)	Output Shape	Param #
dense_315 (Dense)	(None, 64)	86016
dropout_210 (Dropout)	(None, 64)	0
dense_316 (Dense)	(None, 8)	520
dropout_211 (Dropout)	(None, 8)	0
dense_317 (Dense)	(None, 1)	9

Total params: 86,545
Trainable params: 86,545
Non-trainable params: 0

- **Input Layer:** Accepts input with shape (1343,), indicating the model takes a flat vector of 1343 features.
- **Dense Layer:** A fully connected layer with 64 units using ReLU activation.
- **Dropout Layer:** A dropout layer with a rate of 0.5.
- **Dense Layer:** Another fully connected layer following the dropout, this time with 8 units also using ReLU activation.
- **Dropout Layer:** A second dropout layer, now with a rate of 0.3.
- **Dense Layer:** The final layer is a fully connected layer with 1 unit and a sigmoid activation function.

IV) Meta-classifier:

Layer (type)	Output Shape	Param #
p_re_lu_3707 (PReLU)	(None, 56)	56
dense_12901 (Dense)	(None, 8)	456
dropout_4301 (Dropout)	(None, 8)	0
dense_12902 (Dense)	(None, 4)	36
dense_12903 (Dense)	(None, 1)	5

Total params: 553
Trainable params: 553
Non-trainable params: 0

- **Input Layer:** Accepts input with a shape 56 x1 (obtained from the intermediate fusion of three base models).
- **PReLU Layer:** layer with a parametric rectified linear unit activation function.

- **Dense Layer:** A fully connected neural network layer with 8 units. It uses the ReLU activation function. This layer also includes *L2* and *L1* regularization each with a regularization coefficient of 0.05.
- **Dropout Layer:** A dropout layer with a rate of 0.5.
- **Dense Layer:** Another dense layer with 4 units with linear activation.
- **Dense Layer:** The final dense layer with 1 unit and a sigmoid activation function.

Model	#Layers	#Trainable Parameters	#Total Parameters
T5ResConvBiLSTM	6	139746	139746
EmbedCNN	5	29146	29146
PhysicoDNN	3	86545	86545
Meta-Classifier	4 *	553	553

Table S7. Description of the no. of layers and no. of parameters of the proposed models.*Note:*

1. The calculation of the total number of layers in these models excludes the input, lambda, add, dropout, reshape, and flatten layers.
2. *The meta-classifier layer count includes PReLU; however, if PReLU is not considered, the layer count remains 3 (as also stated in the manuscript).

Section S4. Performance Measures

Let **TP** (True Positive) be the count of the predicted Kcr sites, **TN** (True Negative) be the count of correctly predicted non-Kcr sites, **FP** (False Positive) be the count of incorrectly predicted Kcr sites and **FN** (False Negative) be the count of incorrectly predicted non-Kcr sites. Based on these fundamental metrics we define the following measures:

- ***Matthews Correlation Coefficient (MCC)*** = $\frac{(TP \times TN) - (FP \times FN)}{\sqrt[2]{(TP+FP)(TP+FN)(TN+FP)(TP+FN)}}$ (range: [-1,1])
- ***F1*** = $\frac{TP}{TP+0.5(FN+FN)}$ (range: [0,1])
- ***G-mean*** = $\sqrt[2]{\frac{TP}{TP+FN} \times \frac{TN}{FP+TN}}$ (range: [0,1])

  where, $\frac{TP}{TP+FN}$ = Sensitivity (or, True Positive Rate (TPR) or Recall)

  $\frac{TN}{FP+TN}$ = Specificity (or, True Negative Rate (TNR))

- ***Area Under Receiver Operating Characteristic Curve (AUROC):*** Area under the curve of Sensitivity (TPR) plotted against (1- Specificity (TNR) at various decision threshold cut-offs ranging from 0.0 to 1.0. The range of AUROC is [0,1].
- ***Area Under Precision-Recall Curve (AUPR/PrAUC):*** Area under the curve of Precision plotted against Recall (Sensitivity) at various decision thresholds cut-offs ranging from 0.0 to 1.0. The range of AUROC is [0,1].

Section S5. Statistical Significance Testing

We test the statistical significance of LMCrot by performing **McNemar's test**[5,6], a “within-subjects chi-squared test”, for its suitability in paired sample analysis. This test is particularly appropriate for our

study as it is designed to compare the performance of two models on the same dataset, which is the case for LMCrot against the base and existing models in tables 4, 5, 6, and 7 of the manuscript. This test accounts for the correlation between the paired samples, which is a crucial factor in our analysis since the same dataset tests both models. Furthermore, McNemar's test is ideal for binary outcomes, aligning well with our classification tasks. It provides a clear framework to test the null hypothesis (H_0), which states that there is no performance difference between LMCrot and the other models (either base models or existing ones). The alternative hypothesis, conversely, suggests that the prediction performance differs. McNemar's test uses a *2*x*2* contingency matrix based on the predictions from both models under comparison. We calculated the chi-squared ($\mathbf{X}^2$) statistic for each comparison, applying a continuity correction, and set the significance level (a) at 0.05 under the assumption that the null hypothesis (H_0) holds true. The resulting *p*-values, derived from the chi-squared distribution with *L*-1 degree of freedom (here, *L*=2) for each comparison, assist in determining whether to accept or reject the null hypothesis.
Below, the results, including the contingency matrices and *p*-values, are presented for tables 4, 5, 6, and 7 of the manuscript, providing a robust statistical basis for our analysis of LMCrot's performance compared to other models.

i) Comparison of LMCrot with base models on the independent test set (HeLa) (Table 4 in the manuscript)
Below are the contingency matrices obtained from the predictions of LMCrot and base models on the HeLa test set (no. of samples/group: 4842):

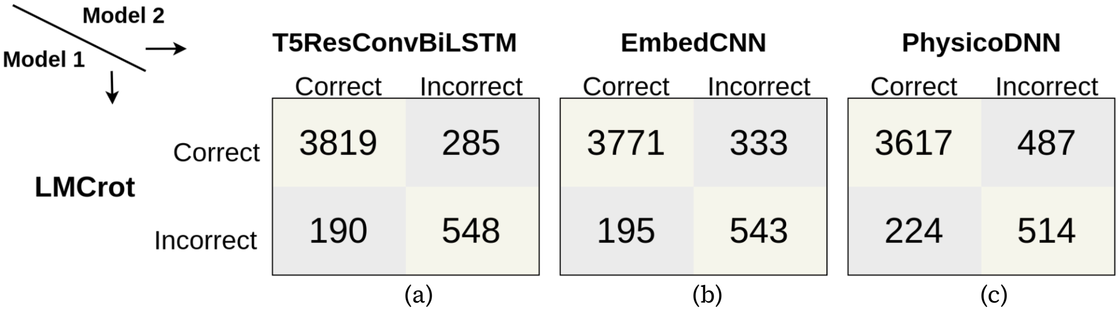

Fig. S1. Contingency matrices obtained from comparing the LMCrot against (a) T5ResConvBiLTM, (b) EmbedCNN, and (c) PhysicoDNN on the HeLa test set.

From the above matrices, we calculated chi-squared ($\mathbf{X}^2$) and thereby *p*-values which are listed in the below table:

Table S8. Chi-squared (*L*=2) and *p*-values obtained by comparing the LMCrot (Model 1) against the three base models (Model 2) on the HeLa test set.

**Models**		**Measures**		**Null Hypothesis (H_0)**
**Model 1**	**Model 2**	**Chi-squared (X^2)**	***p*-value**	
LMCrot	T5ResConvBiLSTM	18.602	1.610e-05	Reject
	EmbedCNN	35.547	2.489e-09	Reject
	PhyscioDNN	96.546	8.721e-23	Reject

From the data presented in Table S8, it is evident that the p-values for all the base models are significantly lower than the chosen significance threshold (α=0.05). This allows us to confidently reject the null hypothesis,

leading to the conclusion that there is a statistically significant difference in the prediction performance between LMCrot and the base models.

Additionally, given that our analysis involves multiple models, we further employed **Cochran's *Q* test**, a more generalized version of McNemar's test. Cochran's Q test is particularly suitable for this scenario as it is designed to test the hypothesis of no difference in performance among multiple classifiers. In this test, the Q statistic is calculated with L−1 degrees of freedom (where L is 4 in this case). Our application of Cochran's Q test yielded a Q value of 271.86 and an extremely small p-value of approximately 1.22e−58. This result strongly suggests that at least one of the models under comparison demonstrates a performance that is significantly different from the others in terms of its predictions. Upon reviewing McNemar's test results in Table S8, it becomes apparent that LMCrot is the model exhibiting this significantly distinct performance.

In summary, both McNemar's test and Cochran's Q test results cohesively point towards LMCrot having a distinctively different and statistically significant prediction performance compared to the base models.

ii) Comparison of LMCrot with DeepCap-Kcr on the independent test set (HeLa) (Table 5 in the manuscript)
Below are the contingency matrix and model comparison table obtained from predictions of LMCrot and DeepCap-Kcr on the HeLa test set (no. of samples/group: 4842):

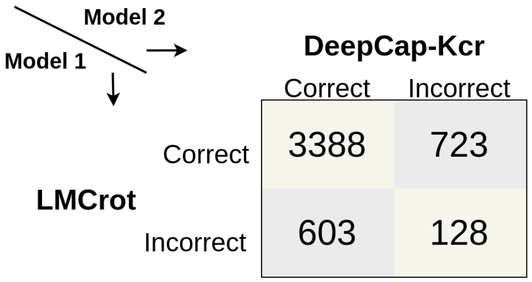

Fig. S2. Contingency matrix obtained from comparing the LMCrot against the DeepCap-Kcr model on the HeLa test set.

Table S9. Chi-squared (L=2) and p-values obtained by comparing the LMCrot (Model 1) against the DeepCap-Kcr model (Model 2) on the HeLa test set.

**Models**		**Measures**		**Null Hypothesis (H_0)**
**Model 1**	**Model 2**	**Chi-squared (X^2)**	***p*-value**	
LMCrot	DeepCap-Kcr	10.679	0.001	Reject

Referring to the results presented in Table S9, it is observed that the p-value is ~0.001, which falls well below the established significance threshold (α = 0.05). This lower p-value leads us to reject the null hypothesis. Consequently, we can conclude that there is a statistically significant difference in the prediction performance between the LMCrot and DeepCap-Kcr.

iii) Comparison of LMCrot with CapsNh-Kcr on the non-histone test set (Table 6 in the manuscript)
Below are the contingency matrix and model comparison table obtained from the predictions of LMCrot and CapsNh-Kcr on the non-histone test set (no. of samples/group: 6682):

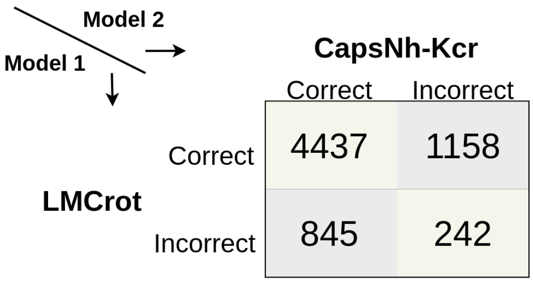

Fig. S3. Contingency matrix obtained from comparing the LMCrot against the CapsNh-Kcr model on the non-histone test set.

Table S10. Chi-squared (L=2) and p-values obtained by comparing the LMCrot (Model 2) against the CapsNh-Kcr model (Model 2) on the non-histone test set.

Models		Measures		Null Hypothesis (H_0)
**Model 1**	**Model 2**	**Chi-squared (X^2)**	***p*-value**	
LMCrot	CapsNh-Kcr	48.599	3.140e-12	Reject

In Table S10, the p-value is less than the significance threshold and thus we reject the null hypothesis. Therefore, there is a significant difference in prediction performance between LMCrot and CapsNh-Kcr.

iv) Comparison of LMCrot with DeepCap-Kcr on the tobacco test set (Table 7 in the manuscript)

Below are the contingency matrix and model comparison table obtained from the predictions of LMCrot and DeepCap-Kcr on the tobacco test set (no. of samples/group:4088):

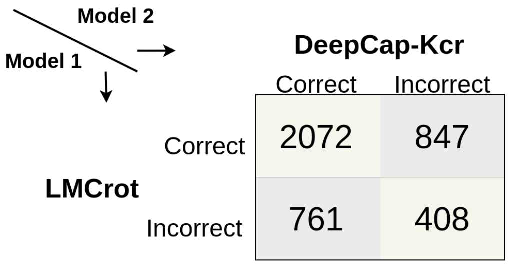

Fig. S4. Contingency matrix obtained from comparing the LMCrot against the DeepCap-Kcr on the tobacco test set.

Table S11. Chi-squared (L=2) and p-values obtained by comparing the LMCrot (Model 2) against the DeepCap-Kcr (Model 2) on the tobacco test set.

Models		Measures		Null Hypothesis (H_0)
**Model 1**	**Model 2**	**Chi-squared (X^2)**	***p*-value**	
LMCrot	DeepCap-Kcr	4.493	0.034	Reject

We reject the null hypothesis since the *p*-value is below the significance threshold. This indicates a significant difference in prediction performance between LMCrot and DeepCap-Kcr on the tobacco test set.

After conducting all of the statistical comparisons, we observed that the LMCrot model demonstrates significant differences from both the baseline models and existing approaches. Thus, the LMCrot model can be considered statistically significant.

Supplementary Results

Table S12. Fivefold cross-validation performance (mean ± one S.D.) of pLMs for different extraction methods namely WSPE, FSPE, WSWE, and FSWE. Among the models evaluated, ProtT5, ProtBERT, ESM-2, and Ankh are transformer-based pLMs, while SeqVec utilizes a BiLSTM architecture.

Extraction Method	Models	MCC	G-mean	F1	AUPR	AUROC
**WSPE**	ProtT5	0.477±0.011	0.737±0.005	0.745±0.008	0.672±0.004	0.813±0.003
	ProtBERT	0.446±0.002	0.720±0.002	0.724±0.014	0.659±0.004	0.794±0.001
	ESM-2	0.470±0.01	0.722±0.005	0.739±0.01	0.665±0.005	0.809±0.004
	Ankh	0.436±0.011	0.709±0.007	0.741±0.005	0.653±0.006	0.793±0.004
	SeqVec	0.457±0.013	0.726±0.007	0.738±0.011	0.662±0.006	0.800±0.005
**FSPE**	ProtT5	0.514±0.004	0.757±0.002	0.760±0.002	0.693±0.002	0.832±0.003
	ProtBERT	0.443±0.014	0.720±0.008	0.725±0.007	0.658±0.008	0.791±0.009
	ESM-2	0.445±0.010	0.720±0.005	0.733±0.009	0.656±0.005	0.799±0.005
	Ankh	0.503±0.014	0.745±0.007	0.757±0.009	0.686±0.007	0.821±0.008
	SeqVec	0.396±0.017	0.697±0.008	0.706±0.011	0.636±0.007	0.766±0.009
**WSWE**	ProtT5	0.592±0.012	0.793±0.007	0.797±0.011	0.733±0.009	0.880±0.004
	ProtBERT	0.581±0.007	0.789±0.003	0.796±0.005	0.724±0.003	0.876±0.005
	ESM-2	0.5556±0.01	0.7786±0.005	0.7817±0.01	0.7127±0.005	0.8663±0.004
	Ankh	0.563±0.015	0.781±0.007	0.786±0.009	0.723±0.008	0.781±0.007
	SeqVec	0.519±0.009	0.756±0.008	0.759±0.015	0.695±0.009	0.846±0.004
**FSWE**	ProtT5	0.614±0.008	0.806±0.004	0.811±0.004	0.743±0.005	0.890±0.004
	ProtBERT	0.609±0.020	0.797±0.011	0.799±0.019	0.737±0.005	0.885±0.008
	ESM-2	0.557±0.007	0.774±0.006	0781±0.010	0.712±0.009	0.864±0.007
	Ankh	0.592±0.007	0.795±0.003	0.800±0.005	0.739±0.006	0.875±0.003
	SeqVec	0.448±0.022	0.722±0.010	0.732±0.017	0.658±0.008	0.798±0.009

Table S13. Fivefold cross-validation performance (mean ± one S.D.) of various ML/DL models for embedding layer, physicochemical properties, and merged representation. The embedding layer utilizes models such as Recurrent Neural Network (RNN), Long Short-Term Memory (LSTM), Bidirectional LSTM (BiLSTM), Convolutional LSTM (ConvLSTM), and Convolutional Neural Network (CNN); for physicochemical properties, Support Vector Machine (SVM), Random Forest (RF), eXtreme Gradient Boosting (XGBoost), AdaBoost (Adaptive Boosting), and Deep Neural Network (DNN) are employed; and the meta-classifier features Logistic Regression (LR), SVM, XGBoost, RF, and Dense Neural Network (DNN).

Representation	Models	MCC	G-mean	F1	AUPR	AUROC
**Embedding layer**	RNN	0.500±0.049	0.745±0.023	0.751±0.020	0.686±0.029	0.840±0.024
	LSTM	0.541±0.008	0.768±0.004	0.779±0.004	0.701±0.005	0.853±0.005
	BiLSTM	0.553±0.024	0.773±0.015	0.784±0.007	0.708±0.017	0.864±0.013
	ConvLSTM	0.563±0.023	0.780±0.011	0.784±0.015	0.716±0.010	0.869±0.010
	CNN(EmbedCNN)	0.585±0.022	0.788±0.013	0.786±0.023	0.733±0.008	0.883±0.005
**Physicochemical**	SVM	0.547±0.011	0.773±0.005	0.773±0.007	0.843±0.004	0.855±0.004
	RF	0.559±0.014	0.778±0.007	0.781±0.008	0.861±0.005	0.854±0.006
	XGBoost	0.560±0.013	0.780±0.006	0.783±0.007	0.879±0.006	0.859±0.005
	AdaBoost	0.534±0.006	0.766±0.003	0.773±0.004	0.851±0.004	0.850±0.003
	DNN(PhysicoDNN)	0.562±0.023	0.780±0.012	0.786±0.011	0.717±0.013	0.861±0.010
**Merged (Meta-classifier)**	LR	0.626±0.014	0.813±0.007	0.813±0.007	0.889±0.003	0.896±0.003
	SVM	0.618±0.003	0.809±0.001	0.809±0.003	0.825±0.004	0.857±0.001
	XGBoost	0.607±0.009	0.804±0.005	0.804±0.005	0.873±0.003	0.884±0.002
	RF	0.610±0.012	0.805±0.007	0.804±0.006	0.870±0.009	0.884±0.006
	DNN (LMCrot)	0.640±0.010	0.819±0.005	0.824±0.006	0.890±0.009	0.898±0.008

Table S14. Fivefold cross-validation performance (mean ± one S.D.) of different fusion methods. In early/input-level fusion, merging occurs at the input layer. Intermediate/feature-level fusion involves merging at an intermediate layer of the models (the final hidden layer in our case). Late/decision-level fusion, on the other hand, is executed at the decision level, integrating outputs towards the end of the process.

Fusion Type	Dimension	MCC	G-mean	F1	AUPR	AUROC
Early	33552 x 1	0.583±0.021	0.787±0.013	0.794±0.013	0.869±0.011	0.877±0.011
Intermediate	56 x 1	0.640±0.010	0.819±0.005	0.824±0.006	0.890±0.009	0.898±0.008
Late	3 x 1	0.620±0.014	0.819±0.011	0.821±0.014	0.883±0.012	0.881±0.010

Note: The dimension of early fusion here is 3352 x 1 (31x 024 from ProtT5, 15x31 from word embedding, and 1343x1 from physicochemical properties).

Table S15. Fivefold cross-validation performance (mean ± one S.D.) of various representation combinations using intermediate fusion.

Feature Combination	MCC	G-mean	F1	AUPR	AUROC
Physicochemical (Phy)	0.562±0.023	0.780±0.012	0.786 ±0.011	0.717±0.013	0.861±0.010
Embedding Layer(Emb)	0.585±0.022	0.788±0.013	0.786±0.023	0.733±0.008	0.883±0.005
ProtT5 (PrtT5)	0.614±0.008	0.806±0.004	0.811±0.004	0.743±0.005	0.890±0.004
PrtT5+Phy	0.620±0.012	0.809±0.006	0.810±0.004	0.882±0.004	0.887±0.002
PrtT5+Emb	0.621±0.021	0.809±0.011	0.812±0.010	0.887±0.007	0.894±0.008
PrtT5+Emb+Phy (LMCrot)	0.640±0.010	0.819±0.005	0.824±0.006	0.890±0.009	0.898±0.008

Supplementary Figures

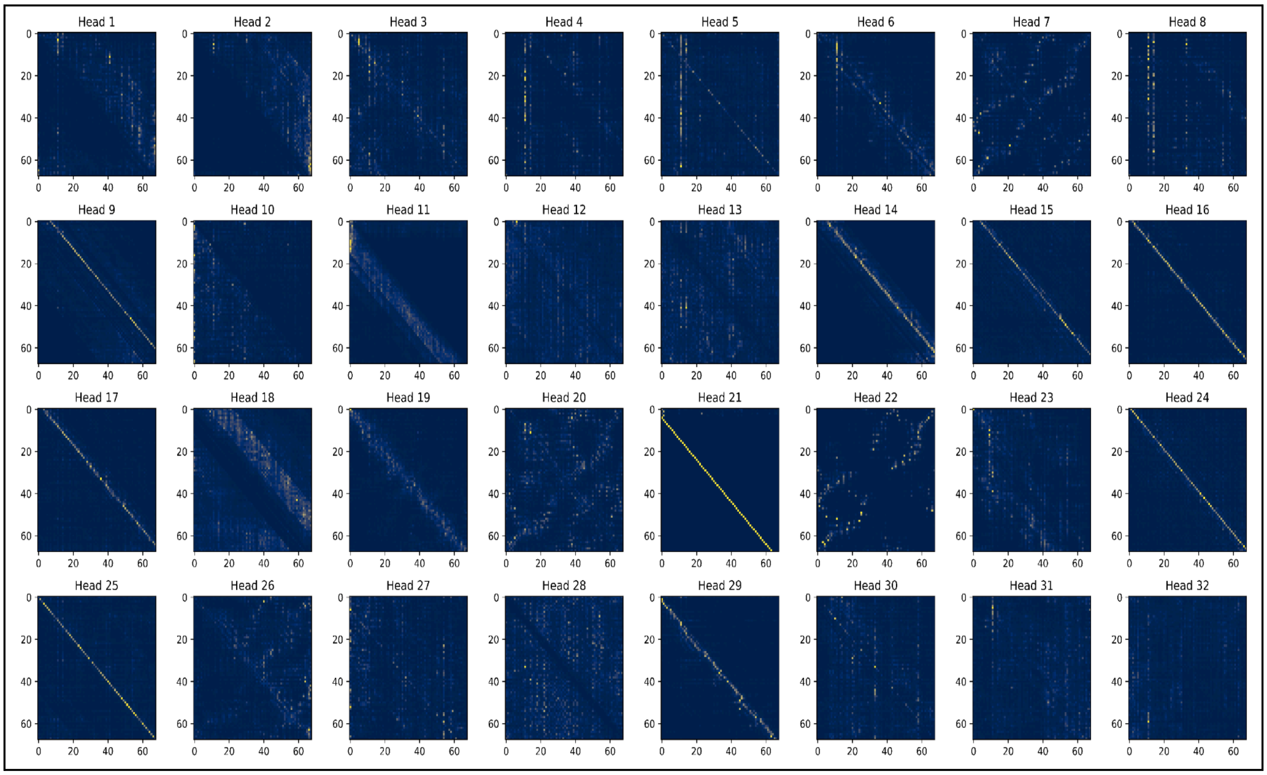

(a)

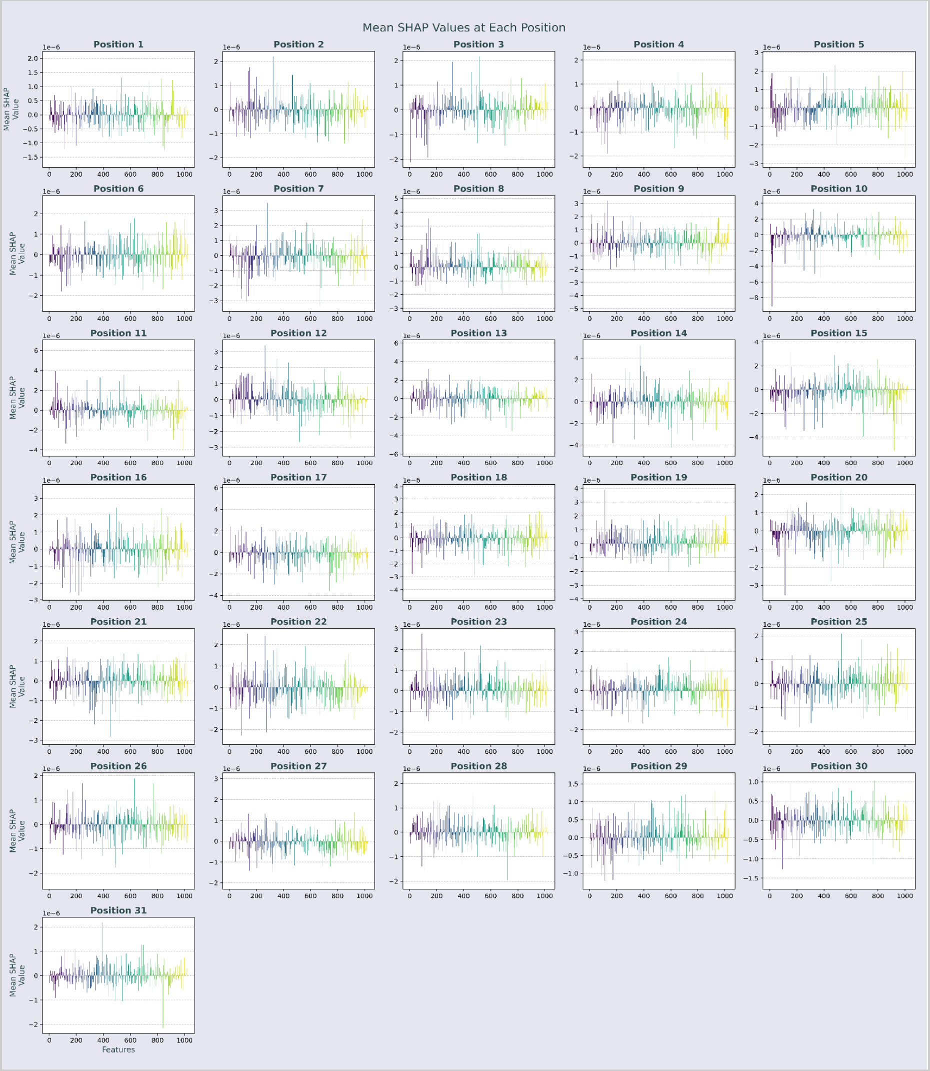

(b)

Figure S5. (a) Heatmap of individual attention heads of the final encoder block of ProtT5 generated for an example protein sequence ' O00244' (length: 68). **(b)** Line plots showing mean SHAP values across all samples of ProtT5 embeddings (dimension=1024) of all 31 residue positions within the window frame.

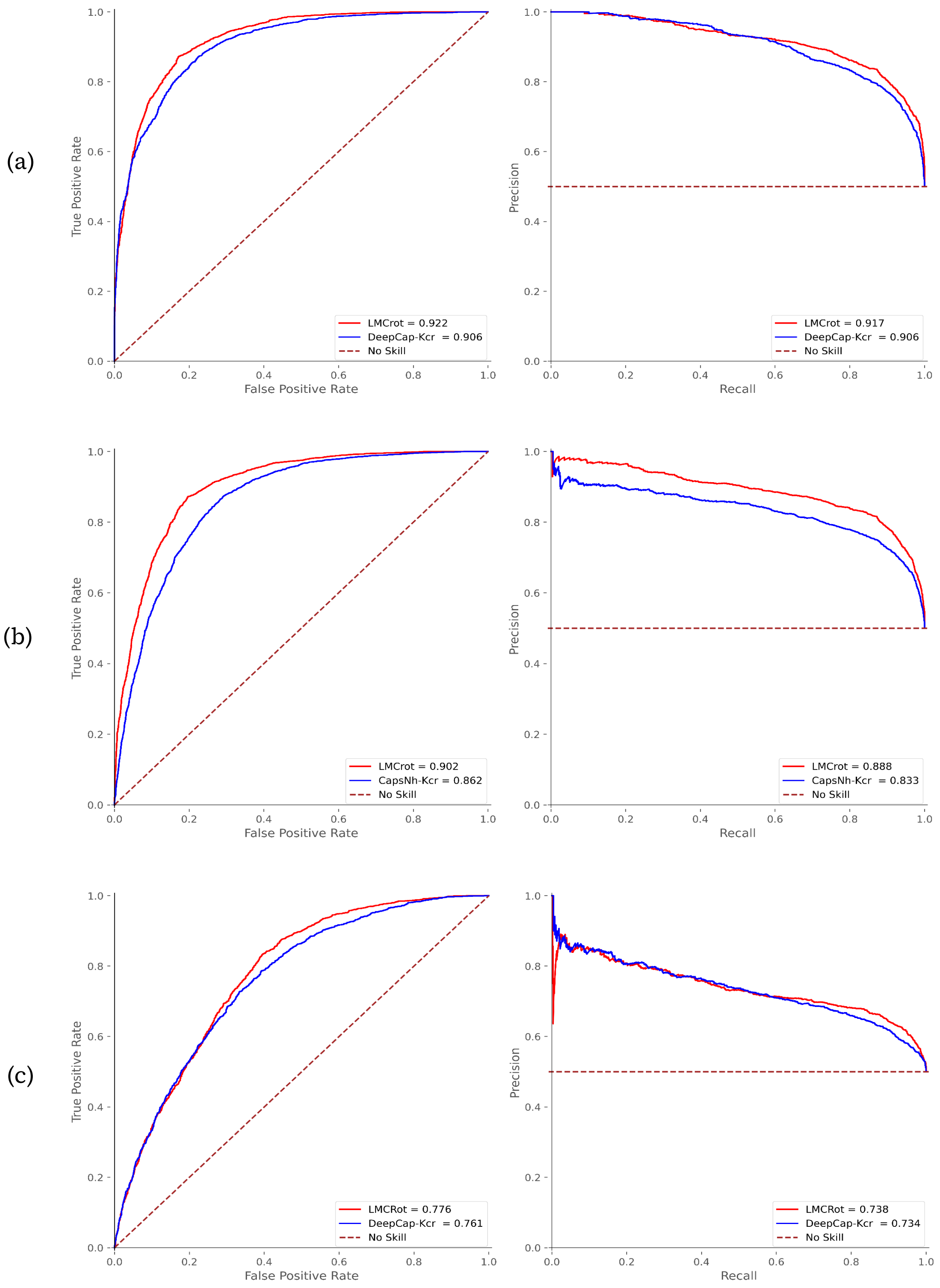

Figure S6. ROC and PR curves comparing LMCrot and **(a)** DeepCap-Kcr on the independent test set (HeLa) (b) CapsNh-Kcr on the non-histone test set **(c)** DeepCap-Kcr on the tobacco test set using cross-species testing.

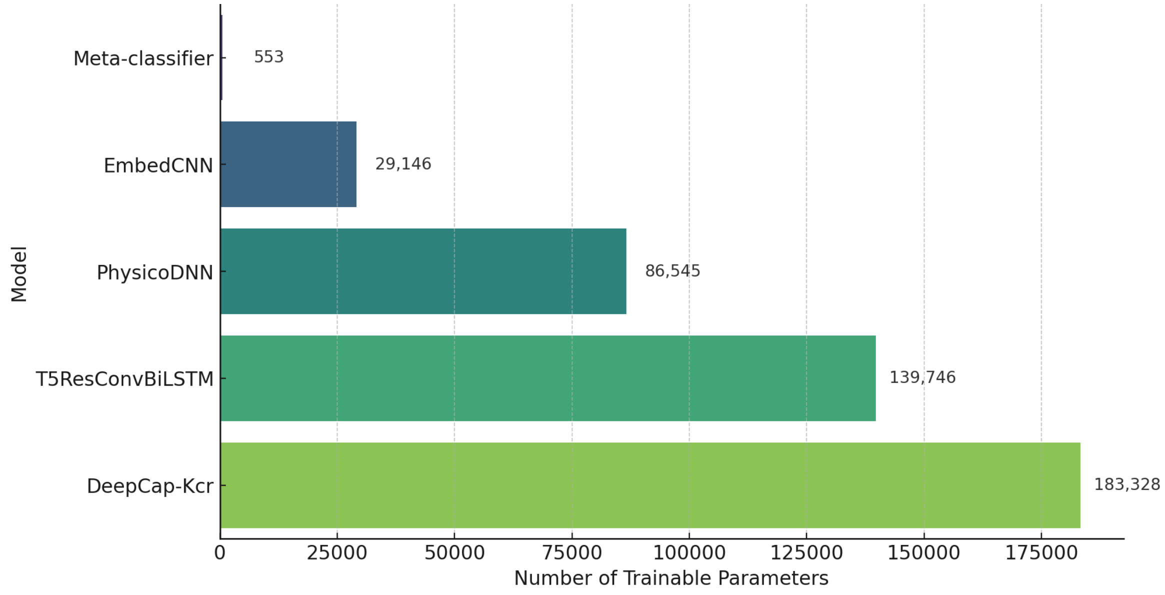

Figure S7. Bar plot comparing the number of trainable parameters of the base models and the meta-classifier with DeepCap-Kcr.

References

[1] Ismail H, White C, Al-Barakati H, Newman RH, Kc DB. FEPS: A Tool for Feature Extraction from Protein Sequence. Methods Mol Biol. 2022;2499:65-104. doi: 10.1007/978-1-0716-2317-6_3. PMID: 35696075.

[2] Darst, B.F., Malecki, K.C. & Engelman, C.D. Using recursive feature elimination in random forest to account for correlated variables in high dimensional data. BMC Genet 19 (Suppl 1), 65 (2018).

[3] Sandberg, M., Eriksson, L., Jonsson, J., Sjöström, M., & Wold, S. (1998). New Chemical Descriptors Relevant for the Design of Biologically Active Peptides. A Multivariate Characterization of 87 Amino Acids. Journal of Medicinal Chemistry, 41(14), 2481–2491.

[4] Waibl F, Fernández-Quintero ML, Wedl FS, Kettenberger H, Georges G, Liedl KR. Comparison of hydrophobicity scales for predicting biophysical properties of antibodies. Front Mol Biosci. 2022 Aug 31;9:960194. doi: 10.3389/fmolb.2022.960194. PMID: 36120542; PMCID: PMC9475378.

[5] Raschka, S. (2018). Model Evaluation, Model Selection, and Algorithm Selection in Machine Learning. ArXiv. /abs/1811.12808.

[6] Janez Demšar (2006). Statistical Comparisons of Classifiers over Multiple Data Sets. Journal of Machine Learning Research, 7(1), 1-30.
